# Are there differences in brain morphology according to handedness?

**DOI:** 10.1002/brb3.730

**Published:** 2017-05-23

**Authors:** Han Jang, Jae Youn Lee, Kang Il Lee, Kang Min Park

**Affiliations:** ^1^ Inje University College of Medicine Haeundae‐gu Busan Korea

**Keywords:** basal ganglia, brain, handedness

## Abstract

**Objective:**

This study aimed to investigate the differences in brain morphology according to handedness.

**Materials and Methods:**

Forty‐two healthy subjects were enrolled (21 right‐handers and 21 nonright‐handers). The two groups were classified according to the Edinburgh Handedness Inventory. Measures of cortical morphology, such as thickness, surface area, volume, and curvature, and the volumes of subcortical structures, such as the amygdala, caudate, hippocampus, globus pallidus, putamen, and thalamus, were compared between the groups according to handedness using whole‐brain 3D T1‐weighted MRI. In addition, we investigated the white matter differences between the groups using diffusion tensor imaging. Moreover, we quantified correlations between the handedness scales of the Edinburgh Handedness Inventory and each measure of different brain morphologies.

**Results:**

The volumes of the right putamen and left globus pallidus in nonright‐handed participants were significantly larger than those who were right‐handed (0.3559 vs. 0.3155%, *p *=* *.0028; 0.1101 vs. 0.0975%, *p *=* *.0025; respectively). Moreover, the volumes of the right putamen and left globus pallidus were negatively correlated with the handedness scales of the Edinburgh Handedness Inventory (*r *= −.392, *p *=* *.0101; *r *= −.361, *p *=* *.0189; respectively). However, the cortex morphology and the other subcortical volumes were not significantly different between the two groups. In addition, we did not find any white matter differences between the groups.

**Conclusions:**

We demonstrated that there were significant differences in brain morphology between right‐handers and nonright‐handers, especially in the basal ganglia, which could produce differences in motor control according to handedness.

## 1 INTRODUCTION

When performing unimanual actions, approximately 90% of the human population prefers using the right hand and only 10% of the population prefers the left hand (Somers, Shields, Boks, Kahn, & Sommer, 2015). This is called handedness, defined as “the individual's preference to use one hand predominately for unimanual tasks and/or the ability to perform these tasks more efficiently with one hand” (Corey, Hurley, & Foundas, 2001). As there are no anatomical differences between the left and the right hand, this difference of preference is believed to originate from differences in brain morphology (Ocklenburg, Garland, Strockens, & Uber Reinert, 2015).

Because dissection and visual inspection is not possible on living brains, researchers conduct studies with noninvasive neuroimaging data such as magnetic resonance imaging (MRI) data. These digital data allow researchers to analyze the brain in depth using advanced statistical software. With these technological advances, we can search for the differences in brain morphology among different groups.

There have been many studies about the relationship between handedness and brain morphology using MRI data. However, many of them focused their attention on only cortical morphology. They have investigated areas including whole gray matter (Good et al., 2001; Ocklenburg, Friedrich, Gunturkun, & Genc, 2016), and individual areas of the cortex such as the central sulcus (Amunts, Jancke, Mohlberg, Steinmetz, & Zilles, 2000), pars triangularis (Foundas, Leonard, & Heilman, 1995), planum temporale (Foundas et al., 1995; Herve, Crivello, Perchey, Mazoyer, & Tzourio‐Mazoyer, 2006), inferior frontal sulcus, precentral sulcus (Herve et al., 2006), and insula (Biduła & Króliczak, 2015; Keller et al., 2011). However, because the cortex is well connected with various subcortical structures and exchanges information with them, morphological investigation of subcortical structures is also important. No previous studies have focused on subcortical structures. In addition, previous studies used voxel‐based morphometry (VBM) for analyzing the cortical morphology, whereas surface‐based analysis (SBA) was rarely used (Amunts et al., 2000; Good et al., 2001; Herve et al., 2006; Kavaklioglu et al., 2017; Ocklenburg et al., 2016). Both VBM and SBA are generally used for analyzing brain morphology. However, as the cerebral cortex has the topology of a 2D sheet and a highly folded geometry, it can be well represented theoretically by SBA, which facilitates the analysis of relationships between cortical regions and provides superior visualization than VBM (Panizzon et al., 2009; Winkler et al., 2010). It has been recently demonstrated that examination of the cerebral cortex using SBA is superior to VBM when assessing brain morphology (Panizzon et al., 2009). Moreover, SBA is attracting more interest because of its subvoxel accuracy and its capability to detect subtle local changes in brain anatomical shapes (Shi & Wang, 2015). Moreover, although it was long thought to be passive tissue, white matter actively affects brain functions, acting as a relay and coordinating communication between different brain regions (Fields, 2008). Although white matter plays an important role in the brain, there have been few studies regarding the differences in white matter between right‐handers and left‐handers (Buchel et al., 2004; McKay, Iwabuchi, Haberling, Corballis, & Kirk, 2016). Diffuse tensor image (DTI) is a commonly used imaging modality to measure the displacement of water molecules across tissue components, which in turn will provide information about the white matter (Madden et al., 2004; Marstaller, Williams, Rich, Savage, & Burianova, 2015).

In this paper, we analyzed the difference in not only cortical structure but also subcortical structure according to handedness, especially using SBA. We also investigated the white matter difference according to handedness using DTI.

## 2 MATERIALS AND METHODS

### 2.1 Measure of handedness

The Edinburgh Handedness Inventory (EHI) is a scale that is used to measure the degree of hand laterality in daily activities. The EHI contains 10 questions concerning writing, drawing, throwing, using scissors, brushing teeth, using a knife, using a spoon, using a broom, striking a match, and opening a box (Annett, 1970; Oldfield, 1971). Participants must report the dominant hand used while doing those activities, and allot either 1 or 2 points for each question according to the degree of dominant hand use. A respondent's score is calculated by (1) subtracting the number of plus signs in the left column from the number in the right column, (2) dividing the difference by the total number of plus signs in both columns, and (3) multiplying the remainder by 100. Scores therefore range from −100 (all plus signs in the left column) to +100 (all plus signs in the right column) (Edlin et al., 2015).

### 2.2 Participants

This study was conducted after receiving the approval of the Institutional Review Board at our institution. It was prospectively conducted in a single tertiary hospital. We enrolled 42 healthy participants. None of them had a history of any neurological or psychiatric disease that could influence brain morphology, and all had a normal MRI on visual inspection. We divided all participants into two groups, right‐handers or nonright‐handers. The right‐handers were defined as participants who scored 100 in the EHI, whereas the nonright‐handers were those who scored <100 in the EHI. We collected demographic characteristics such as sex and age at the time of MRI.

### 2.3 MRI data acquisition

All subjects underwent a brain MRI. All scans were conducted on a 3.0 T MRI scanner (AchievaTx, Phillips Healthcare, Best, the Netherlands) equipped with an eight‐channel head coil. All individuals received conventional brain MRI protocols, including axial and coronal 2D T2‐weighted images, which were obtained with turbo spin‐echo sequence [repetition time (TR)/echo time (TE) = 3000/80 ms, slice thickness = 5 mm, echo train length = 14, field of view (FOV)  = 210 mm, matrix size = 512 × 512], and axial and coronal 2D T1‐weighted images, which were obtained with inversion recovery sequence [inversion time (TI)  = 800 ms, TR/TE = 2000/10 ms, slice thickness = 5 mm, echo train length = 7, FOV = 210 mm, and matrix size = 512 × 512]. 3D T1‐weighted images were obtained with turbo field echo sequence with the following parameters: TI = 1300 ms, TR/TE = 8.6/3.96 ms, Flip angle (FA)  = 8°, and 1 mm^3^ isotropic voxel size. To obtain data more quickly, SENSE (SENSitivity Encoding) parallel imaging with an acceleration factor of 2 was adapted. The DTI was performed using spin‐echo single shot echo‐planar pulse sequence with a total of 32 different diffusion directions (TR/TE = 8620/85 ms, FA = 90°, slice thickness = 2.25 mm, acquisition matrix = 120 × 120, FOV = 240 × 240 mm^2^, and b‐value = 1000 s/mm^2^).

### 2.4 MRI data processing and analysis using FreeSurfer and FSL software

The FreeSurfer image analysis software version 5.1 (http://surfer.nmr.mgh.harvard.edu/) was installed on a CentOS. The processing flow of FreeSurfer consisted of several steps as follows: volume registration with the Talairach atlas, bias field correction, initial volumetric labeling, nonlinear alignment to the Talairach space, and final labeling of the volume. Then, the cortical surface of each hemisphere was inflated to an average spherical surface to locate both the pial surface and the WM/GM boundary. The subcortical structures (hippocampus, amygdala, caudate, putamen, globus pallidus, and thalamus) were split automatically on the basis of T1‐weighted images using FreeSurfer. The split regions were used to compare volume. We measured cortical morphological characteristics such as thickness, surface area, volume, and curvature using FreeSurfer's Qdec application, and statistical significance was defined as *p *<* *.05 with multiple correction and family wise error.

To perform tract‐based spatial statistics (TBSS) analysis, all raw DTI data were preprocessed with FSL (http://www.fmrib.ox.au.uk/fsl). First, eddy current distortions and head motions were corrected by spatially normalizing all the diffusion‐weighted images. Subsequently, skull‐stripping was applied to exclude nonbrain tissues and regions. Finally, we computed the diffusion tensor and scalar measures, including fractional anisotropy (FA) and mean diffusivity (MD). FA and MD were analyzed using protocols provided by TBSS. We normalized individual FA volumes of the two groups to the MNI template space through affine registration. The aligned FA images were averaged to yield a mean FA image and then thinned to create the FA skeleton of the mean FA image. The skeleton was regarded as representing the common tract pattern of all participants from the two groups. Then, the FA threshold (0.2) was set on the skeleton to exclude gray matter and cerebrospinal fluid from the final analysis. Each subject's FA image was projected onto the skeleton. The significance threshold for between‐group differences was set at *p *< .05 (FWE‐corrected for multiple comparisons) using the threshold‐free cluster enhancement option in the “randomize” permutation‐testing tool in FSL (5000 permutations). Regional FA differences were then localized according to the probabilistic Johns Hopkins University White Matter Atlas. Group comparisons of MD images were performed similarly.

### 2.5 Statistical analysis

We used the chi‐square test for analyzing whether there was a difference in the sex of the participants according to handedness. We used the Student's *t* test for analyzing whether there was a difference in the age of the participants according to handedness and for investigating the volume differences in subcortical structures, such as the amygdala, caudate, hippocampus, globus pallidus, putamen, and thalamus, between right‐handers and nonright‐handers. When analyzing the volume differences in six subcortical structures, a *p*‐value <.004 (0.05/12, Bonferroni correction) was set as statistically significant. In addition, the correlation between the handedness scales of EHI and each measure of different brain morphologies was analyzed by Spearman's rank correlation coefficient. We used MedCalc^®^ (MedCalc software version 13, Ostend, Belgium) to analyze statistics.

## 3 RESULTS

There were no significant demographic differences between right‐ and nonright‐handers, including sex and age (Table 1). The median score of the EHI was 100 in right‐handers (21 subjects with 100 of EHI score), whereas that of the EHI was 40 in nonright‐handers (eight subjects with EHI score ≥50 to <100, five subjects with EHI score ≥0 to <50, six subjects with EHI score ≥−50 to <0, one subject with EHI score >−100 to <−50, one subject with 100 of EHI score).

**Table 1:** A comparison of demographic characteristics between right‐handers and nonright‐handers

Variable	Right‐handed (*n *= 21)	Nonright‐handed (*n *= 21)	*p*‐value
Age, mean (years)	32.6	32.6	1.0000
Male, *n* (%)	12 (57.1)	10 (47.6)	.7574
EHI, median	100	40	<.001

EHI, Edinburgh handedness inventory.

**Figure 1:**
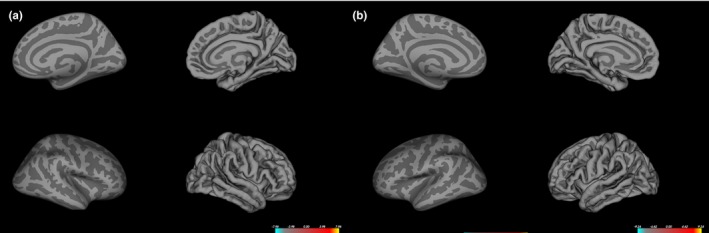
The differences in cortical morphology between right‐handers and nonright‐handers in the right (a) and left (b) hemisphere. No significant differences in cortical morphology are found (multiple corrections with *p *<* *.05)

Regarding cortical morphologies such as thickness, surface area, volume, and curvature, there were no differences between the two groups (Figure 1).

**Figure 2:**
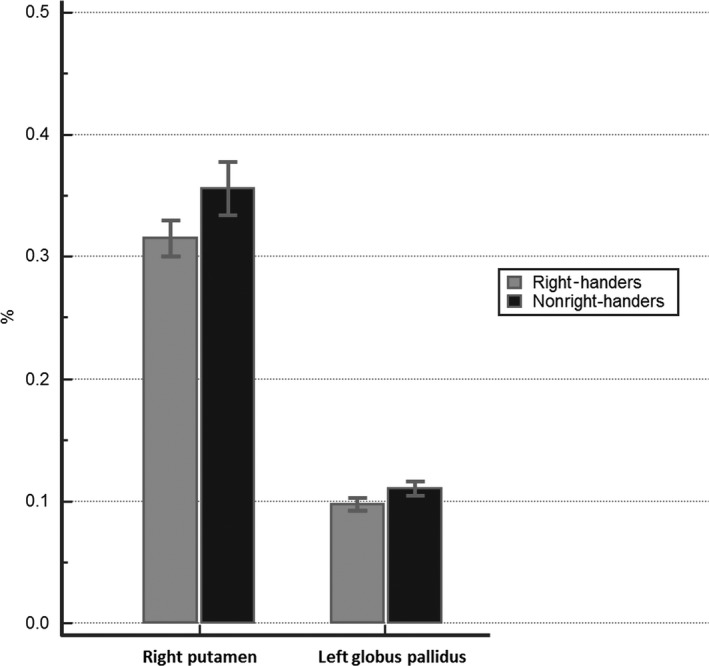
A comparison of volumes of putamen and globus pallidus between right‐handers and nonright‐handers. These figures indicate that the right putamen and left globus pallidus of nonright‐handers are significantly larger than those of right‐handers

**Figure 3:**
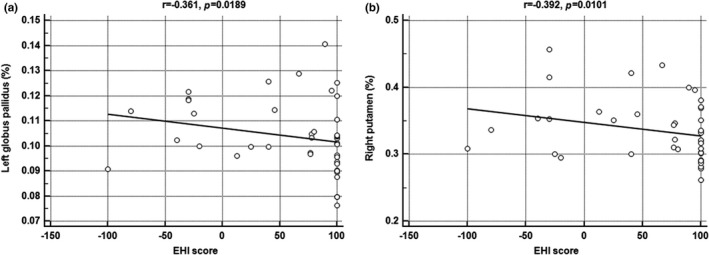
Negative correlation between Edinburgh handedness inventory (EHI) score and volume of (a) left globus pallidus, and (b) right putamen. These figures indicate that the volume of each structure decreases as the EHI score increases

In the measurement of volume of subcortical structures, there were statistical differences in the right putamen and left globus pallidus (Table 2) (Figure 2). The right putamen and left globus pallidus of nonright‐handers were significantly larger than those of right‐handers (0.3559 vs. 0.3155%, *p* = .0028; 0.1101 vs. 0.0975%, *p* = .0025; respectively). In addition, there was a significantly negative correlation between the EHI score and each volume of the left globus pallidus and right putamen (*r *= −.361, *p* = .0189; *r *= −.392, *p* = .0101) (Figure 3). There were no significant differences in other subcortical structures such as the right and left amygdala, hippocampus, caudate nucleus, thalamus, right globus pallidus, and left putamen.

**Table 2:** A comparison of volumes of subcortical structures between right‐handers and nonright‐handers

Structure	Right‐handers (*n *= 21)	Nonright‐handers (*n *= 21)	*p*‐value
Left structures (%)			
Amygdala	0.0981	0.1113	.0094
Caudate	0.2265	0.2432	.0894
Globus pallidus	0.0975	0.1101	.0025^a^
Hippocampus	0.2775	0.3039	.0181
Putamen	0.3413	0.3825	.0071
Thalamus	0.5420	0.5590	.3951
Right structures (%)			
Amygdala	0.1031	0.1092	.2766
Caudate	0.2070	0.2280	.0374
Globus pallidus	0.0977	0.1064	.0418
Hippocampus	0.2857	0.3055	.1404
Putamen	0.3155	0.3559	.0028^a^
Thalamus	0.4754	0.5051	.1602

EHI, Edinburgh handedness inventory.

a *p *<* *.004.

Regarding the white matter analysis using DTI, there were no differences in the FA and MD values between the two groups.

## 4 DISCUSSION

The main finding of our study was that the basal ganglia in nonright‐handers were larger than those in right‐handers. There are three major points that provide strength to our study compared to previous studies. First, we analyzed MRI data, especially using SBA, which has better sensitivity in finding subtle morphological differences than VBM. Second, we investigated the relationship between handedness and subcortical structures, which have been rarely studied. Finally, we also studied the differences in white matter using DTI according to handedness. In addition, these outcomes will be very important findings for brain morphology analysis in patients with neurological disease. We demonstrated that healthy subjects had different brain morphology according to handedness from the present study. Therefore, we need correction for handedness when investigating brain morphology in patients with neurological disease.

Basal ganglia, such as the putamen and globus pallidus, play an important role in motor control (Chakravarthy, Joseph, & Bapi, 2010; Stocco, Lebiere, & Anderson, 2010). Because the putamen is connected with many other brain structures that control body movement, it is believed that the putamen is involved in motor performance, motor preparation, specifying amplitudes of movement, and movement sequences (Alexander & Crutcher, 1990; DeLong, Alexander, et al., 1984; Delong, Georgopoulos, et al., 1984; Marchand et al., 2008). In addition, the globus pallidus is known for its function in the regulation of voluntary movement. It is involved in the constant subtle regulation of movement that allows people to walk, talk, and engage in a wide variety of other activities with minimal level of disruption (Brotchie, Iansek, & Horne, 1991; Stephenson‐Jones et al., 2016). In a previous study, older adults performed poorly in motor control and skill acquisition tasks, which were related to volume loss of basal ganglia (Chalavi et al., 2016).

From the results of the present study, we suggest that left‐handers have better motor control than right‐handers because of their large volume of basal ganglia. There have been some studies that are in agreement with our suggestion. First, it was reported that top athletes have a large volume of basal ganglia, and many of them were left‐handed. Moreover, the volumes of globus pallidus in top professional diving athletes were much larger than those in a nonathletic group (Zhang et al., 2013). Second, the rate of left‐handers among top wrestlers who won medals was greater than the rate of right‐handers, irrespective of gender (Ziyagil, Gursoy, Dane, & Yuksel, 2010). Furthermore, there was a study reporting that left‐handedness was an intrinsic advantage in cricket games. An analysis of cricket yearbooks showed that there were a relatively high proportion of professional cricketers who bowled left‐handed (Wood & Aggleton, 1989). Conversely, despite these results, there are some controversies about the high rate of left‐handers in top athletes. A previous study analyzed the distribution of left‐handed interactive sports players and noninteractive sports players. They found that the rate of left‐handers was higher in interactive sports players than in noninteractive sports players (Grouios, Tsorbatzoudis, Alexandris, & Barkoukis, 2000). They hypothesized that the better motor control of left‐handers could be due to their low proportion in the world so that right‐handers cannot maneuver properly in interactive sports. Third, the ability of left‐handers when using their right hands was slightly better than the ability of right‐handers when using their left hands (Judge & Stirling, 2003). This phenomenon could be caused by the large volume of basal ganglia in left‐handers, resulting in overall better motor control. Fourth, another study compared the motor and sensory conduction velocity of the median nerve between right‐ and left‐handers. The study found that the sensory conduction velocity of the median nerve in left‐handers was significantly faster than that of right‐handers, despite the fact that there was not much difference in the motor conduction velocity of the median nerve (Gupta, Sanyal, & Babbar, 2008). This difference in conduction velocity might contribute to the difference in motor control according to handedness.

In addition, there have been other studies supporting our results. It is well known that there is a significant correlation between the volume loss of the temporal lobe and cognitive decline of patients with Alzheimer's disease (Cho et al., 2014). However, there was also evidence that the volume loss of the putamen was associated with cognitive decline of patients with Alzheimer's disease. In addition, another study found that left‐handers who had Alzheimer's disease were less affected by cognition decline than right‐handers. Thus, because left‐handers had larger volumes of putamen than right‐handers, left‐handers might be less vulnerable to cognitive decline in Alzheimer's disease.

Several studies investigated the relationship between handedness and cortical morphology (Amunts et al., 2000; Biduła & Króliczak, 2015; Foundas et al., 1995; Good et al., 2001; Herve et al., 2006; Keller et al., 2011; Ocklenburg et al., 2016). Especially, recent researches indicated that the structural asymmetry of the insular cortex was significantly related to the lateralization of gesture and language (Biduła & Króliczak, 2015; Keller et al., 2011). However, no significant differences in cortical morphologies including insular cortex between right‐ and nonright‐handers were detected from the present study. This finding may be caused by a relatively small number of subjects, which may be a limitation of this study. However, we found statistically significant differences in the volumes of basal ganglia between right‐ and nonright‐handers even with this small sample size and multiple corrections, which could be interpreted as indicating statistically very meaningful results.

Another limitation of this study was that we defined both left‐handers and ambidextrous individuals as nonright‐handers. In addition, because it was so difficult to only enroll left‐handers who score −100 in EHI, we enrolled the subject with EHI score ≥50 to <100 into the group of nonright‐handers. In Korea, the number of left‐handers is especially low (Gupta et al., 2008). In addition, we had to enroll age‐ and sex‐matched participants. Thus, we also analyzed the differences in volumes of basal ganglia (right and left putamen and globus pallidus) between the 21 subjects with EHI score of 100 and the 13 subjects with EHI score ≥−100 to <50 to confirm our findings, again (when analyzing the volume differences in four subcortical structures, a *p*‐value <.013 (0.05/4) was set as statistically significant). It also revealed that the right putamen and left globus pallidus of nonright‐handers were significantly larger than those of right‐handers (0.3550 vs. 0.3155%, *p *=* *.0091; 0.1112 vs. 0.0975%, *p *=* *.0119; respectively), which findings were consistent with the results of present study.

In conclusion, we demonstrated that there were significant differences in brain morphology between right‐handers and nonright‐handers, especially in the basal ganglia. This morphological difference may produce differences in motor control according to handedness.

## CONFLICT OF INTEREST

The authors declare no potential conflict of interests.
